# Epidemiology of Psoriasis and Comorbid Diseases: A Narrative Review

**DOI:** 10.3389/fimmu.2022.880201

**Published:** 2022-06-10

**Authors:** Jin Bu, Ruilian Ding, Liangjia Zhou, Xiangming Chen, Erxia Shen

**Affiliations:** ^1^Hospital for Skin Disease (Institute of Dermatology), Chinese Academy of Medical Sciences and Peking Union Medical College, Nanjing, China; ^2^Sino-French Hoffmann Institute, School of Basic Medicine, The Second Affiliated Hospital, Guangzhou Medical University, Guangzhou, China; ^3^The State Key Laboratory of Respiratory Disease, The First Affiliated Hospital, Guangzhou Medical University, Guangzhou, China

**Keywords:** psoriasis disease, comorbid disease, epidemiology, autoimmune chronic diseases, prevalence

## Abstract

Psoriasis is a chronic autoimmune inflammatory disease that remains active for a long period, even for life in most patients. The impact of psoriasis on health is not only limited to the skin, but also influences multiple systems of the body, even mental health. With the increasing of literature on the association between psoriasis and extracutaneous systems, a better understanding of psoriasis as an autoimmune disease with systemic inflammation is created. Except for cardiometabolic diseases, gastrointestinal diseases, chronic kidney diseases, malignancy, and infections that have received much attention, the association between psoriasis and more systemic diseases, including the skin system, reproductive system, and oral and ocular systems has also been revealed, and mental health diseases draw more attention not just because of the negative mental and mood influence caused by skin lesions, but a common immune-inflammatory mechanism identified of the two systemic diseases. This review summarizes the epidemiological evidence supporting the association between psoriasis and important and/or newly reported systemic diseases in the past 5 years, and may help to comprehensively recognize the comorbidity burden related to psoriasis, further to improve the management of people with psoriasis.

## Introduction

Psoriasis is a common chronic inflammatory disease with prevalence of 0.33%-0.6% in different races (1, 2), and affects around 125 million people worldwide. With the understanding of the biological nature of psoriasis, it has been recognized as an autoimmune disease with significant impacts on health implications extending beyond the skin (3).

The first extracutaneous comorbid disease of psoriasis was diabetes, which was reported in 1897 (4), and research on the association between psoriasis and systemic comorbidities has rapidly grown in past decades, mainly focusing on cardiometabolic diseases, chronic kidney disease (CKD), gastrointestinal diseases, malignancy, mood disorders, infection, and psoriatic arthritis (PsA), which was summarized in a comprehensive review published in 2017 (5). In the past 5 years, increasing epidemiologic evidence supports that more systemic diseases are identified as extracutaneous comorbidities of psoriasis. In this review, we summarized these data to improve comprehensive understanding of the burden of comorbid diseases associated with psoriasis, which may be essential for future medical management for patients with psoriasis.

## Materials and Methods

A search was performed on PubMed using the following search strategy: psoriasis AND ((comorbid disease) OR comorbidity) AND (epidemiology OR incidence) between 2016 and 2021. In total, 1874 papers were collected, 1160 of them were removed because of irrelevance and 489 were removed because of non-original article type; finally 216 original articles published were included in the current review.

## Cardiovascular Diseases

The incidence of overall atherosclerotic cardiovascular disease is higher in psoriasis compared with controls (2), and the prevalence of atherosclerotic cardiovascular disease varies in different races, as it is 2.4-fold higher in African American than in white patients with psoriasis (1). Psoriasis is independently associated with myocardial infarction along with hypertension, dyslipidemia, and diabetes mellitus (6), in both sexes in adults (2, 6), whereas the risk for ischemic stroke is increased in women with moderate to severe psoriasis (2).

Many studies on the association between cardiovascular diseases and psoriasis have been concluded in at least one meta-analysis (**Table 1**) (7–13), including coronary artery disease (CAD) (such as stroke, myocardial infarction, and cardiovascular death), atrial fibrillation, and aortic aneurysm. Two meta-analyses specially investigated the risk of cardiovascular diseases according to the disease severity (7, 11). Two systematic reviews checked the associations between psoriasis and cardiovascular comorbidities in pediatric populations, one confirmed the existence of the association (12), however the other one found a null association (13).

**Table 1:** Summary of systematic review and meta-analyses assessing the association between psoriasis and cardiovascular diseases.

Study	Study dates	Total number of patients		Number of studies included	Outcomes	Composite measure of association (95% CI)
		Psoriasis	No psoriasis			
Raaby et al. (7) 2017	Up to mid-summer 2015	NR	NR	13 high-quality observational studies	CAD risk, including stroke, myocardial infarction, cardiovascular death	Stroke: HR=1.10 (1.0–1.19) in mild patients; HR=1.38 (1.20–1.60) in severe patients Myocardial infarction: HR=1.20 (1.06–1.35) in mild patients; HR= 1.70 (1.18–2.43) in severe patients Cardiovascular death: HR=1.37 (1.13–1.67) in severe patients
Zhao et al. (8) 2018	From inception to March 2018	464	10,408	6 observational studies	Risk of CAD diagnosed by angiography	OR= 1.42 (0.81–2.47)
Kaiser et al. (9)2019	2000- 30 May 2018	1,427	9,670	14 cross-sectional observational and case-control studies	Prevalence and burden of CAD using CCS and CCTA	CAD risk: RR=1.14 (1.04–1.26; *P*=0.004), RR=1.71 (1.28–2.30; *P* < 0.001) for more severe CAD (CCS >100). Risk of coronary plaques identified by CCTA: RR=1.77 (1.37–2.28; *P* < 0.001).
Upala et al. (10) 2017	From inception to November 2015	12,755	4,561,838	4 observational studies	Atrial fibrillation risk	Pooled HR=1.42 (1.22–1.65); No difference between mild [HR=1.22 (1.15–1.30)] and severe [HR=1.51 (1.22–1.87)] psoriasis
Yu et al. (11) 2020	From inception to 20 July 2019	24,864	5,681,661	3 cohort studies	Aortic aneurysm Risk	HR=1.30 (1.10-1.55, I2 = 53.1%). No difference on risk between severe psoriasis [HR=1.51 (1.04-2.19)] and mild psoriasis [HR=1.24(1.08-1.42)]. Not statistically increased risk in female patients [HR=1.55, (0.65-3.72)], patients ≥50 years old [HR=4.05, (0.69-23.75)], and patients with diabetes [HR=0.97(0.83-1.14)]
Phan et al. (12) 2020	1993-2019	43,808	5,384,057	17 including 10 prospective case-control study and 7 retrospective analysis	Cardiometabolic risk in children	Overweight/obesity OR=1.58 (1.14-2.19)/OR 2.45 (1.73-3.48); association with obesity is dependent on the severity of disease [OR =1.66 (1.16-2.37), *P* = 0.005] Waist: height ratio >0.5 OR= 1.87 (1.12-3.13); Diabetes OR=2.32 (1.34-4.03); Hypertension OR= 2.19 (1.62-2.95); Hyperlipidemia OR= 2.01 (1.66-2.42); Metabolic syndrome OR =1.75 (1.75-7.14); Ischemic heart disease or heart failure OR=3.15 (1.06-9.42).
Badaoui, et al. (13)	2009-2016	11,787 (7,660 children and 4,127 adults)		16 articles: 8 retrospective case–control studies, 4 retrospective national registry studies, 2 retrospective studies, 2 cross-sectional studies.	Risk of metabolic and cardiovascular comorbidity in children Influenceon metabolic and cardiovascular comorbidity in adulthood	A higher risk of overweight and obesity in children with psoriasis No higher risk of hypertension, diabetes, dyslipidemia, metabolic syndrome, and major cardiovascular events. Age at onset of psoriasis did not increase the frequency of comorbidity in adulthood.

CAD, coronary artery disease; CCS, coronary calcium score; CCTA, cardiac computed tomography angiography; HR, hazard ratio; OR, odds ratio; N/A, not applicable; NR, not reported.

Psoriasis with chronic inflammation promotes development of pulmonary embolism, and is related to a cardiovascular and venous thromboembolism risk, but lower in-hospital mortality (14). Independent predictors for venous thromboembolism in psoriasis patients included older age, diabetes mellitus, and corticosteroid usage (15). Psoriasis was an independent predictor for gastro-intestinal bleeding (14). A cohort study in Sweden suggested that low cardiorespiratory fitness at an early age was associated with an increased risk of psoriasis and PsA in men (16).

Several clinical and biological variables including anti-HDL antibodies (17), coronary flow reserve (18), lymphocyte ratio (19), total plaque area, and carotid intima-media thickness (20) have been used for evaluation or prediction of CAD in psoriasis. Inflammation (high-sensitivity C-reactive protein) played an important role in the development of visceral adipose tissue and its effect on early atherogenesis (21). Noncalcified coronary burden risk was associated with high-sensitivity C-reactive protein (21) and serum high-sensitivity troponin-T (22). Machine learning methods were successfully applied to identify noncalcified coronary burden predictors in psoriasis patients by coronary computed tomography angiography (23). Inflammasome signaling is correlated with severity of psoriasis disease, proinflammatory endothelial transcripts, and circulating interleukin (IL)-6 (24). A systematic review and meta-analysis including 24 studies found that psoriasis patients had a higher homocysteine level [standardized mean differences (SMD)=0.41, 95% CI:0.21-0.61] and a lower folate level (SMD=-0.94, 95% CI: -1.49 to -0.40) in serum compared with controls (25). These above findings provide a better evaluation on the heightened risk of cardiovascular disease in patients with psoriasis.

Treatment modalities on psoriasis may affect cardiovascular comorbidities of psoriasis. A systematical review including 14 studies confirmed that weight loss can improve the psoriasis area and severity index (PASI) score of patients, and prevent the onset of psoriasis in obese individuals (26). Adalimumab showed anti-inflammatory effects and improved flow-mediated dilation, and fumaric acid esters interacted favorably with the cholesterol metabolism (27). Cyclosporine and mixed conventional systemic treatments were associated with increased major adverse cardiovascular events risk, and the cumulative of major adverse cardiovascular events incidence in the phototherapy and biologic was lower, while methotrexate was not associated with major adverse cardiovascular events (28).

## Metabolic Diseases

Metabolic diseases are usually considered as high-risk factors for cardiovascular diseases, and the studies focusing on the association between psoriasis and cardiovascular risk factors are increasing. Most studies have been summarized in at least one meta-analysis (**Table 2**), and cardiovascular risk factors include obesity measured by body mass index (BMI), waist circumference (WC), waist-to-hip ratio, weight (29), type 2 diabetes (30), and metabolic syndrome (MetS) of adults (31) and children (32).

**Table 2:** Summary of systematic review and meta-analyses assessing the association between psoriasis and metabolic diseases-cardiovascular disease risk factors.

Study	Study dates	Total number of patients		Number of studies included	CV risk factor	Composite measure of association (95% CI)
		Psoriasis	No psoriasis			
Aune et al. (29) 2018	Up to 8 August 2017	17,636	695,471	7 prospective studies	Adiposity risk	Summary RR 1.19 (1.10–1.28) for a 5-unit increment in BMI; 1.24 (1.17–1.31) per 10 cm increase in WC; 1.37 (1.23–1.53) per 0.1-unit increase in waist-to-hip ratio; 1.11 (1.07–1.16) per 5 kg of weight gain.
Friis et al. (30) 2019	1952-2016	1,508	1,452	26 clinical studies	Type 2 diabetes risk	Evidence is not unequivocally supporting common pathophysiological denominators in psoriasis and type 2 diabetes
Rodríguez-Zúñiga et al. (31) 2017	January 1980 to January 2016	25,042	131,609	14 observational studies including case-control, cross-sectional, or cohort) with 156,651 participants	MetS risk	A pooled OR= 1.42 (1.28-1.55) OR for prospective studies=1.52 (1.27-1.76); OR for retrospective studies=1.38 (1.19-1.57) OR, 1.76 (0.86-2.67) for Middle Eastern (in Israel, Turkey, and Lebanon) OR, 1.40 (1.25-1.55) for European studies (in Germany, Italy, the United Kingdom, Norway, and Denmark) Systemic treatment reduced risk for MS (OR, 1.37(1.23; 1.50)
Pietrzak et al. (32).2017	1966 to June 2015	965 children	NR	7	MetS risk	OR = 6.10 (2.66–13.98)
Wu et al. (33) 2020	2009-2018	862	NR	6 studies	Body weight and BMI increase risk in patients receiving biologics	Treatment of TNF-α inhibitors was associated with an increase in body weight (mean difference 1.40 kg, 95% CI: 0.88-1.93 kg) and BMI (0.39 kg/m^2^, 95% CI: 0.24-0.54 kg/m^2^).
Zou et al. (34) 2021	Inception to 1 May 2020	448	377	11 studies	Association between serum visfatin levels and (1) psoriasis and (2) the severity of psoriasis	Significantly higher levels of visfatin than the controls SMD=0.90 (0.52, 1.28) Serum visfatin levels were associated with ethnicity, PASI and BMI. Visfatin levels were correlated with PASI r=0.51(0.14, 0.75)

TNF, tumor necrosis factor; PASI, Psoriasis Area and Severity Index; BMI, body mass index; NR, not reported.

RR, relative risk; BMI, body mass index; HDL, high-density lipoprotein; WC, waist circumference; CI, confidence interval.

### Obesity/Overweight

A nationwide prospective cohort study in Korea found that metabolically unhealthy non-obese subjects and metabolically unhealthy obese subjects had a higher risk of psoriasis compared to metabolically healthy non-obese subjects (35).

A meta-analysis including seven prospective studies concluded that adiposity as measured by BMI, WC, waist-to-hip ratio, and weight gain was associated with increased risk of psoriasis (29). WC measuring central adiposity is a specific factor affecting psoriatic risk (36), after adjustment for confounders and BMI (37). Two Mendelian randomization analyses using genetic variants as instrumental variables confirmed that higher BMI causally increased the risk of psoriasis (38, 39).

A systematic review and network meta-analysis including six studies concluded that compared with conventional systemic treatments, tumor necrosis factor (TNF) α inhibitors were associated with a significant increase in body weight, while anti-IL-12/23 or anti-IL-17 biologics had no effect on an increase of body weight or BMI (33).

### Metabolic Syndrome

The prevalence of MetS in patients with psoriasis ranges from 14.3% to 50% (31, 40, 41). The strength of these associations between psoriasis and MetS has been repeatedly confirmed by several observational studies (40, 42–44), and psoriatic patients had at least a double risk of MetS compared with non psoriatic individuals (41). The psoriasis risk tended to increase with the number increase of MetS components, and this trend was evident in obese subjects (44). A systematic review and meta-analysis of 14 observational studies found that a greater risk for MetS was reported in Middle Eastern than European patients (31). However, in Thailand, no significant association was found between MetS and psoriasis severity using PASI (45).

Significant differences were found in relation to the prevalence of cardiovascular risk factors, MetS, and major cardiovascular events in patients with psoriasis compared to non-psoriatic population, however, differences were not seen among psoriasis severity groups (43). After adjusting for all other MetS factors, WC and blood pressure remained significantly associated with noncalcified coronary burden (46).

Women with MetS have a higher chance of being psoriatic (47). Prevalence of MetS (32) and insulin resistance (48) was significantly higher in children with psoriasis. In most studies included in a meta-analysis conducted by Pietrzak et al. (32), a significantly decrease of high-density lipoprotein cholesterol level was found in children with psoriasis.

The increase in expression of surviving and heat shock protein 27, heat shock protein 60, and heat shock protein 90, and the decrease in cyclin D1 expression may be an important molecular mechanism involved in the development of MetS in psoriasis (49). Selenoprotein P was increased in psoriasis and significantly decreased after psoriatic treatment (50).

IL-17A monoclonal antibody treatment cannot only ameliorate psoriatic lesions but also restore dysregulation of lipid metabolism in psoriasis patients (51). MetS negatively affects psoriasis severity and treatment outcomes (45).

### Type 2 Diabetes

A systematic review including 26 clinical studies concluded that the available literature does not unequivocally support the association between psoriasis and type 2 diabetes because the low quality of studies included (30). However psoriasis was reported to be associated with type 2 diabetes, independent of visceral fat (52), and psoriasis severity is an independent risk factor of the homeostatic model assessment of insulin resistance (53). Lee et al. (54) firstly confirmed the association between psoriasis and diabetic complications including diabetic retinopathy and end-stage renal disease (ESRD). The effect of age seemed to differ between psoriasis sexes, men from 35 to 49 years old were at a lower risk of type 2 diabetes compared to their peers of 75 years of age and older, whereas women of 50 to 64 years old were at an increased risk of type 2 diabetes (55).

### Lipid Metabolism

Psoriasis was significantly associated with hypercholesterolemia, hospital-diagnosed hypertension (56), and hyperlipidemia (57). The accumulating evidence of the nature of psoriasis and the risk of cardiovascular comorbidities is potentially due to an acquired hypercoagulability (58). Psoriasis significantly increased the risk of CKD, and so did hyperlipidemia. Statin treatment for hyperlipidemia reduced the CKD risk in psoriasis patients compared to treatment without statins (57).

A meta-analysis including 11 studies demonstrated that psoriasis patients had higher levels of visfatin (SMD, 0.90; 95% CI, 0.52-1.28), which was associated with ethnicity, PASI, and BMI (34). Visceral fat was associated with psoriasis, hyper-triglyceridemia, low high-density lipoprotein, and type 2 diabetes, and these associations were linked to serum IL-6, adiponectin, tumor necrosis factor, and insulin resistance (52).

### Autoimmune Thyroid Disease

A meta-analysis suggests that thyroid peroxidase antibody positivity (pooled OR, 1.71, 95% CI: 1.27-2.31), hypothyroidism, and hyperthyroidism (pooled OR, 1.17, 95% CI: 1.03-1.32) might be associated with prevalent psoriatic disease (59). Patients with thyroid dysfunction have significantly higher PASI scores and elevated serum C-reactive protein levels than those without thyroid dysfunction (60). In Taiwan, the psoriasis group had an increased risk for incident hyperthyroidism, Graves’ disease, hypothyroidism, and Hashimoto thyroiditis compared with controls (61), and a high prevalence of Hashimoto’s thyroiditis is especially observed in women with psoriasis (62).

### Gout

A nationwide population-based cross-sectional study in Taiwan found gout was associated with psoriasis (adjusted OR, 1.30, 95% CI:1.20-1.42) (63), and a positive correlation was found between PASI scores and serum uric acid levels in psoriasis patients (64).

## Mental Health Diseases

A great deal of literature was produced to assess different aspects of psychology in psoriasis. The hazard ratio (HR) of any mental disorder is 1.75 (95%CI, 1.62-1.89) in psoriasis persons compared with the general population, and the main mental disorders reported were as follows: vascular dementia, schizophrenia, bipolar disorder, unipolar depression, generalized anxiety disorder, and personality disorders (65). In both sexes, psoriatic patients and their partners suffer from psychopathological and sexual consequences related to disease severity (66). A significant increase in psychiatric disorders occurs in pediatric psoriasis, with a 6.65-fold greater risk of depression and a 9.21-fold greater risk of anxiety, compared with the controls (67).

Risk factors of distress include female gender, a younger age of disease onset, those with self-assessment of severe psoriasis (68), type D personality (69), younger patients, and those with lesions on sensitive or visible areas (70). Alcohol disorders, not illicit drug use, are more common in patients with psoriasis (68).

An association was observed between the severity of psoriasis and mood disturbances with an impact on quality of life (71), and men tended to have a shorter time to onset for most mental health disorders than women, except for neurotic disorders and anxiety disorders (72), which may influence the appropriate management of male patients.

The risk of mental disorders increased among psoriasis individuals who had completed short-term education compared with those with medium and long-term education (65), while Zhang et al. (73) concluded there was no significant difference in psychological health between psoriasis patients with different levels of educational attainment. Except for anxiety and depression, psoriasis patients suffer from social distress and social avoidance.

### Depression/Anxiety

Psoriasis and depression might have a bidirectional association (74). The prevalence of anxiety/depression among psoriasis patients was 11.52%-27.00% (75–77). Psoriasis patients were at an increased risk for depression (78–81), anxiety disorders (79–81), anxiety and depression co-occurrence (81), and somatoform disorders (79) compared with the referent cohort. Risk factors in psoriasis patients associated with depression were: 20-50 years (77), female sex (77, 82), major comorbid diseases (77, 82), and low income (77, 81). Patients with moderate-to-severe psoriasis had a significant risk of depression and somatoform disorders compared to patients with mild disease (79), and the highest risk was observed among patients with severe psoriasis aged 40-50 years (78). However a HUNT3 study found depressive symptoms do not seem to be a major concern among individuals with psoriasis (83).

Major depressive disorder is a risk factor for the development of psoriasis (84, 85), especially in the male sex (85). Somatic and anxiety symptoms, as well as BMI, are closely linked to dermatology-related quality of life (86).

A unique study conducted in a South East Asian population determined that Indian ethnicity was a predictor of depression (*P* = 0.024) (87), and it provided invaluable insight into predictive factors of adverse effects of psoriasis on distress in a special population.

A systematic review described that the prevalence of anxiety in patients with psoriasis was significantly higher than healthy controls (OR: 2.91, 95% CI: 2.01-4.21), and an improvement in anxiety symptoms with therapy of psoriasis was demonstrated (**Table 3**) (88).

**Table 3:** Summary of systematic review and meta-analyses assessing the association between psoriasis and mental health diseases.

Study	Study dates	Total number of patients		Number of studies included	Outcomes	Composite measure of association (95% CI)
		Psoriasis	No psoriasis			
Fleming et al. (88) 2017	2001-2015	938,194 participants		15 studies including 8 cross-sectional studies, 4 cohort studies, and 2 randomized control trials control studies, and 1 case–control study	Anxiety risk	HR=1.29-1.31, P = 0.001, OR=2.91, 95% CI, 2.01-4.21
Singh et al. (89) 2017	1946-2017	330,207	1,437,376	18	Suicides risk	Suicidal ideation a pooled OR=2.05 (1.54-2.74) Suicidal behaviors a pooled OR=1.26 (1.13-1.40)
Pompili et al. (90) 2021	Databases inception to February 2020	624,593	12,252	21	Suicides risk	Suicidal ideation OR = 1.97 (1.26-3.08) Suicidal acts OR = 1.42(1.05-1.92)
Chi et al. (91) 2017	Databases inception to 24 March 2017	381,431	1,072,178	5	Suicides risk	Suicide RR=1.13 (0.87-1.46), (1) Suicide attempt RR=1.25(0.89-1.75), (2) Suicidality RR=1.26 (0.97-1.64)
Charoenngam et al. (92) 2021	From inception to 12 July 2019	740,454 from 5 studies	10,013,063 from 5 studies	6 cohort studies	Dementia risk	Pooled RR=1.16 (1.04–1.30)
Yen et al. (93) 2021	Up to 12 July 2019	10 studies included a total of 16,574 psoriasis cases; 1 study evaluated 7,118 patients with dementia	10 studies included 45,078 controls; 1 study included 21,354 controls.	11	1. Risk of dementia or cognitive impairment 2. Psoriasis risk	9 of the 11 included studies found a significant positive association between the two diseases, one study a null association, and one study an inverse association.
Snast et al. (94) 2018	1964-2015	32,537 patients	NR	39 studies including 19 surveys, 7 cross-sectional studies, 12 case–control studies and 1 cohort study. 5 study for meta-analysis	Psoriasis risk Stress disorder risk Evaluating stressful events prior to psoriasis exacerbation Association between stress levels and exacerbation of psoriasis	Onset of psoriasis a pooling OR=34, 95% CI:18–64; Stress disorder risk OR=12, 95% CI: 08–18. Association between stress levels and exacerbation of psoriasis r = 028, P < 005 More frequent/severe preceding events among patients with psoriasis.
Stewart et al. (95) 2018	January 1987–December 2016	19,617 in total		12 studies including 2 epidemiological cross-sectional, 3 cohort, and 7 case-control studies; Due to heterogeneity of data, a meta-analysis could not be conducted	Onset and/or recurrence of psoriasis. Association between severity of psychological stress and severity of psoriasis.	A probable temporal association between different measures of psychological stress and onset, recurrence, and severity of psoriasis.
Gupta et al. (96) 2016	Finalized on 13 January 2015	54,827	NR	33	Formal sleep disorders risk: OSA; restless legs syndrome; insomnia (chronic insomnia and transient insomnia)	Prevalence of OSA is 36%-81.8% in psoriasis versus 2%-4% in the general population. increase risk of restless legs syndrome prevalence of 15.1%-18% in psoriasis versus 5%-10% in European and North American samples. The prevalence of insomnia is 5.9%-44.8% in psoriasis vs. 10% prevalence of chronic insomnia and 30-35% prevalence of transient insomnia in general population (P>0.05)

OSA, obstructive sleep apnea; OR, odds ratio; CI, confidence interval; NR, not reported.

For moderate-to-severe psoriasis, unadjusted incremental all-cause healthcare costs associated with anxiety/depression were $8,077. Psoriasis treatments that improve psychiatric symptoms may reduce their economic burden (76). Therefore, screening for depressive/anxiety can lead to an increase of utilization of mental health care and improvement of psoriasis.

### Suicides

Two systematic reviews and meta-analyses found more suicidal ideation and acts with psoriasis (**Table 3**) (89, 90), and severe psoriasis and younger age were risk factors (89). However, another systematic review and meta-analysis with five population-based cohort studies found no increase in the risk of suicide, suicide attempt, or suicidality among people with psoriasis (**Table 3**) (91).

Because the systematic review and meta-analyses from Pompili et al. (90) analyzed the association between suicidal risks with not only psoriasis but also atopic dermatitis, Matterne et al. (97) compared the two systematic reviews and meta-analyses with opposite conclusions (89, 91) focusing on suicidal risks with psoriasis, and found that the differences mainly came from the data source. Chi et al. (91) only included cohort studies while Singh et al. (89) included cross-sectional, case-control, and cohort studies. Chi et al. (91) included five high quality studies according to the Newcastle-Ottawa Scale. Singh et al. (89) included 18 studies that were defined as to be medium quality to high quality.

The results of the association between suicides and psoriasis in later studies are still contradictory: the risk of suicidal behavior in individuals with psoriasis was confirmed in Canada (97), but not in Taiwan (98).

A systematic review and meta-analysis identified no increased risk for depression, anxiety, or suicidality with treatment of secukinumab in a pooled data analysis from 10 studies in patients with moderate-to-severe plaque psoriasis (99).

### Dementia

Two systematic review and meta-analyses summarized the association between dementia and psoriatic disease (**Table 3**) (92, 93), however two population-based studies in Taiwan found contradictory results on the association between psoriasis and dementia (100, 101). Phototherapy and systemic treatment might not have a protective effect against dementia in psoriatic patients (100).

### Stress

Psychological stress plays an important role in the development of psoriasis, but the details of this association remain to be clearly defined. A systematic review demonstrates a temporal relationship between stress and psoriasis (**Table 3**) (95). A tertiary level of education was an independent risk factor while a higher monthly income was a protective factor for psoriasis populations (87). However, another systematic review and meta-analysis including 39 studies concluded there is no convincing evidence that preceding stress is significantly associated with exacerbation/onset of psoriasis because data are primarily based on retrospective studies with nonnegligible limitations (**Table 3**) (94).

Cohort studies from the UK and Denmark observed no evidence supporting increased long-term risk of psoriasis following bereavement (102).

### Sleep Disorders

A systematic review observed an overall 36% to 81.8% prevalence of obstructive sleep apnea in psoriasis (96), and psoriasis is also associated with restless legs syndrome, which is a possible sign of autonomic activation in psoriasis (103).

### Fatigue

Psoriasis was associated with an elevated risk of chronic fatigue syndrome, which is differentiated by sex and age (104). Fatigue severity was associated with smoking, pain, and depression, but not with psoriasis severity (105).

### Newly Reported Mental Comorbidity of Psoriasis

An increased risk of Parkinson disease (106) and migraine, especially migraine with aura (107) was reported in patients with psoriasis; while a higher risk of psoriasis was found in patients with schizophrenia, and Th17 and pro-inflammatory cytokines may act as a link between these two diseases (108). There is a relationship between psoriasis and alexithymia (109) and dental fear (110), and impulse control proved to be the strongest predictor to psoriasis disability (111).

A meta-analysis showed significant, small-to-medium effects of psychosocial interventions on quality of life (0.28, 0.04-0.51) and anxiety (0.36, 0.15-0.57), but a not significant effect on depression (0.37, -0.05-0.80) (112).

## Nervous System Disease

Multiple sclerosis (MS) is an autoimmune-mediated inflammatory disease of the central nervous system, characterized by myelin and various degrees of axonal loss.

Three systematic review and meta-analyses concluded that psoriasis is associated with an increased risk of MS (113), and vice versa (114), especially for family members of those with MS (115) (**Table 4**).

**Table 4:** Summary of systematic review and meta-analyses assessing the association between psoriasis and MS and vitiligo.

Study	Study dates	Total number of patients		Number of studies included	Outcomes	Composite measure of association (95% CI)
		Psoriasis	No psoriasis			
Islam et al. (113) 2019	1 January 1990 to 1 November 2017	NR	48,832 MS patients	11 observational studies	MS risk	RR=1.607 (1.322–1.953)
Liu et al. (114) 2019	July 2018	43,643	1,097,374	11 studies (5 case-control, 4 cross-sectional and 2 cohort studies)	Psoriasis risk	OR=1.29 (1.14-1.45) HR=1.92 (1.32-2.80)
Charlton et al. (115) 2019	Inception to 8th August 2018	NR	NR	5 studies	Psoriasis risk in family members of MS patients	OR=1.45 (1.07, 1.97)
Yen et al. (116) 2019	On 22 January 2018	120,866	79,907 vitiligo	10 case control studies	Vitiligo risk	Summary OR vitiligo risk in psoriasis: 2.29 (1.56–3.37), psoriasis risk in vitiligo: 3.43 (1.86–6.33)

NR, not reported; OR, odd ratios; RR, relative risk; HR, hazard risk; MS, multiple sclerosis.

One study evaluated that the RR of MS risk was 0.184 in mild psoriasis (95% CI 1.46–2.30) and 2.61 in severe psoriasis (95% CI 1.44– 4.74) (117). A significant association between psoriasis and MS was detected after adjusting for variables of age, sex, PsA, and exposure of TNF-α agent (118).

## Gastrointestinal Disease

### Inflammatory Bowel Diseases

IBDs include two chronic idiopathic inflammatory diseases: ulcerative colitis (UC) and Crohn’s disease (CD), which are the main gastrointestinal comorbidities of psoriasis. Two meta-analyses found significant bidirectional associations between psoriasis and IBDs, presence of CD (OR 2.0, 95% CI 1.4-2.9) and UC (OR 1.5, 95% CI 1.2-2.0) was significantly associated with psoriasis (119), and psoriasis had an increased risk of CD (RR= 2.53; 95% CI, 1.65-3.89) and UC (RR=1.71; 95% CI, 1.55-1.89) (120). Psoriasis patients were reported to have a higher prevalence of gastrointestinal signs and symptoms (feeling full/bloated, belly pain, diarrhea, mucus/blood in stool, and unintentional weight loss) (121), and sex and PsA were particular risk factors (122). Psoriasis-UC patients may have a higher BMI and milder skin symptoms than those with psoriasis alone (123), while psoriasis-CD patients have a mild (early-onset) psoriasis but a severe CD phenotype (124). While a cohort study in Denmark found a limited incremental effect of IBDs on risk of comorbidities in patients with psoriasis (125).

Association of psoriasis/PsA with IBD influences management strategy (126), and young patients and those with severe psoriasis may require comprehensive management (127). Time to CD, but not UC, diagnosis was significantly longer for patients with psoriasis compared with the general controls, and patients with systemic treatment had the longest time to CD and UC (122). A systematic review on 132 randomized, controlled, double-blinded studies concluded that infliximab and adalimumab had demonstrated efficacy in psoriasis, PsA, UC, and CD (128).

### Non-Alcoholic Fatty Liver Disease

The prevalence of NAFLD in psoriasis patients is high and related to a higher prevalence of MetS, bacterial translocation, and a higher pro-inflammatory state (TNF-α, TGF-β level, and bacterial translocation) (129). Early onset of psoriasis was independently associated with greater odds of NAFLD, hypertriglyceridemia, hyperuricemia, and smaller odds of diabetes compared to late onset (130). The NAFLD fibrosis score proved a high significant correlation (*P*<0.0001) with fibrosis histological lesions in psoriatic patients (131). Enhanced liver fibrosis score and procollagen-3 N-terminal peptide are elevated in psoriasis and PsA, and enhanced liver fibrosis score may be superior to procollagen-3 N-terminal peptide alone (132).

### Other Liver Diseases

A two-way meta-analysis including 18 studies found bidirectional associations between celiac disease and psoriasis; the OR is 2.16 (95% CI, 1.74-2.69) for celiac disease in patients with psoriasis and 1.8 (95% CI, 1.36-2.38) for psoriasis in patients with CD (133). The prevalence of gallstones was higher in the psoriasis patients (adjusted OR is 1.18, 95% CI: 1.14-1.23) (134). Patients with psoriasis may have an elevated risk for diverticulitis, not appendicitis or cholecystitis, compared to the general population (135). Psoriasis is associated with cirrhosis, even among patients without systemic therapy (136).

## CKD

Psoriasis and CKD might have a bidirectional association. Psoriasis has a significantly increased risk of CKD (57), and is weakly associated with CKD stages 3-5 (137). The cumulative incidence of psoriasis was higher in CKD patients with anemia than those without, and low hemoglobin levels were significantly related to the risk of psoriasis in CKD patients (138). Statin treatment for hyperlipidemia reduced the CKD risk in patients with psoriasis (57).

In Korea, psoriasis was associated with the risk of ESRD, and the risk of developing ESRD in patients with psoriasis differed according to the type of treatment (with acitretin or not) and the presence of arthritis (139). A retrospective cohort study from Taiwan reported a higher HR for psoriasis in hemodialysis patients than that of the control group, and age <60 years was a risk factor of psoriatic development (140).

Patients with moderate-to-severe psoriasis had a significantly increased risk for development of IgA nephropathy and glomerular disease (141).

## Malignancy

A systematic review and meta-analysis including 112 cohort studies found the overall prevalence of cancer in psoriasis patients was 4.78% (95% CI, 4.02%-5.59%), with an RR of 1.21 (95% CI, 1.11-1.33) (142). There was an increased risk of the following cancers in psoriasis patients: keratinocyte cancer, lymphomas, lung cancer, bladder cancer (142), and melanoma or hematologic cancer (143). A prospective longitudinal cohort study showed that a higher standardized incidence ratio of cancer was observed in women compared to men (144). Another systematic review and meta-analysis including 58 cohort and case-control studies found severe psoriasis was associated with a mortality risk of cancer (overall RR, 1.22; 95% CI, 1.08-1.38) (145). However, the included studies had a high level of heterogeneity, which made interpretation challenging.

There is concern that systemic treatment of psoriasis may increase the risk of cancer, however, the effects of systemic treatment including biologics on malignancy are contradictory. Psoriasis patients receiving systemic treatment were reported to have a significantly elevated HR for cancers of bone and cartilage (146), non-Hodgkin lymphoma, and non-melanoma skin cancer (147). However, a systematic review and meta-analysis concluded that no increased risk of cancer was found (RR, 0.97; 95% CI, 0.85-1.10) in psoriasis patients treated with biologics (142), even basal cell carcinoma (BCC) or squamous cell carcinoma (SCC) in patients with a history of BCC or SCC (148), since a history of previous BCC or SCC is by far the strongest predictor of future BCC and SCC. Increased prescribing of acitretin and decreased prescribing of narrowband UVB were clearly evident in psoriasis patients with a history of BCC or SCC (148). The slightly increased risk of cutaneous SCC does not seem to be associated with methotrexate, but rather with disease severity, other anti-psoriatic treatments, and ultraviolet exposure (149). There were no differences in malignancy risk among topical treatments, systemic agents, phototherapy, or biologics, and psoriasis patients with cancer did not have worse survival than patients without psoriasis (143).

## Infection

Psoriasis is associated with an unremarkable increase in the risk of serious infection (fully adjusted HR is 1.36, 95% CI 1.31–1.40) (150), and psoriasis severity is a predictor of serious infection risk (151).

### COVID‐19

COVID‐19 is the worst epidemic in the last 2 years, and psoriasis patients might discontinue treatment without consulting a dermatologist due to fear of SARS-CoV-2 infections, especially patients who require systemic therapy. However, a Danish study reported that most patients felt to a great extent well treated (67.0%) and safe about their treatment in general (76.4%) (152). A retrospective cohort study in Brazil suggested that systemic therapy did not worsen COVID-19 in psoriasis (150). It is not necessarily concluded that TNFi is safer than biologics targeting IL-17 and IL-23 or not, with respect to risk of respiratory tract and SARS-CoV-2 infections (153). In patients with moderate-to-severe psoriasis, nonbiologic systemic therapies were associated with a higher risk of COVID-19-related hospitalization than with biologic use, and established risk factors included older age, male sex, non-white ethnicity, and having comorbidities (154). A so-called “cytokine storm syndrome” may increase the risk of mortality in COVID-19 patients (155). Although, there are no obvious contraindications to the use of inactivated vaccines, evaluating the risk-benefit ratio of maintaining ongoing immunosuppressive therapy before performing the vaccine is mandatory (156).

### Hepatitis B and C

A large US population study reported the prevalence of chronic hepatitis B/C in psoriasis and non-psoriasis patients was 0.5%/0.8% and 1.3/1.6%, respectively, and there was no significant difference between the populations with and without psoriasis in terms of prevalence of hepatitis B or C (157). A higher prevalence of hepatitis C virus was demonstrated in adults with psoriasis and a higher rate of hepatic decompensation in hepatitis C virus+ individuals with moderate-severe psoriasis (158). Long-term use of methotrexate may not be associated with a liver cirrhosis risk among psoriatic patients with chronic viral hepatitis (159). Upregulation of inflammatory cytokines in patients with hepatitis viral infection possibly increases susceptibility to developing psoriasis (160).

### HIV

Infection of HIV was an independent risk factor for development of psoriasis (adjusted HR, 1.80; 95%CI: 1.38-2.36) (161), and severe psoriasis was an independent risk factor for steatosis in patients with HIV infection (OR, 12; 95% CI, 1.2-120) (162). Treatment of psoriasis patients with HIV is a challenge as immunosuppressive agents may reactivate or induce infection in such patients, so biologics become a possible effective choice in psoriatic patients with a stable HIV infection, and it is also mandatory to monitor CD4 count and HIV viral load, in order to avoid possible causes of infection (163).

### Mycobacterium

Single nucleotide polymorphism of rs1465788 in the zinc finger protein 36 ring finger protein-like 1 gene was identified as an early-onset psoriasis risk gene which demonstrates opposite associations with leprosy and psoriasis in a Chinese Han population (164).

Detection of latent *Mycobacterium tuberculosis* infection is mandatory before biotherapy in psoriasis as biotherapy may reactivate the infection. The national investigation of latent tuberculosis infection in Italian patients with psoriasis reported a prevalence of 8.5%, and independent risk factors included male sex, age over 55 years, and being entered into conventional treatment (165). The heparin-binding hemagglutinin, a higher sensitivity marker in response to a mycobacterial antigen, may help to prioritize patients who receive prophylactic interventions of *Mycobacterium tuberculosis* before starting biotherapies (166).

### Staphylococcal Colonization

A systematic review and meta-analysis found that psoriatic patients faced an increased risk for colonization of staphylococci compared to healthy controls, and the pooled prevalence was 39.2% (95% CI 33.7-44.8) *vs.* 35.3% (95% CI 25.0-45.6) (167). A 4.5-fold increase of *Staphylococcal aureus* colonization was found on the patients’ skin compared to healthy controls, and 60% was in the nares (167).

### Herpes Zoster

A Taiwanese cohort study confirmed psoriasis was associated with an increased risk of HZ (adjusted HR of 1.29, 95% CI= 1.07-1.56), which involved differences in sex and age (168). The risk of HZ was significantly increased among the moderate to severe psoriasis group and was associated with immunosuppressive therapy (151). Although systemic therapy may play an important role in the risk of HZ, the intrinsic factors of psoriasis should be considered.

## Musculoskeletal System

### PsA

Almost 1/3 of patients with psoriasis develop PsA, which showed strong associations with psoriasis (adjusted OR:10.08; 95% CI: 7.97-12.74) (169). A meta-analysis including 266 studies found that the overall pooled proportion of PsA among adult psoriatic patients was 19.7%, and was 3.3% (95% CI, 2.1%-4.9%) in adolescents <18 years. The PsA prevalence was 22.7% (95% CI, 20.6%-25.0%), 21.5% (95% CI, 15.4%-28.2%), 19.5% (95% CI, 17.1%-22.1%), 15.5% (95% CI, 0.009%-51.5%), and 14.0% (95% CI, 11.7%-16.3%) in psoriasis from European, South American, North American, African, and Asian patients respectively (170). However, the high heterogeneity of studies included may have affected the estimates.

Incidence of PsA was 1.48, 3.00, and 5.49 per 100 patient-years in mild, moderate, and severe psoriasis patients, respectively (171). Risk of PsA increases steadily with duration of cutaneous symptoms following psoriasis onset (172, 173). PsA patients have a larger clinical burden, characterized by higher comorbidity rates, than those with psoriasis. Ratios of psoriasis-PsA comorbidity rates relative to psoriasis-only ranged from 1.1 for allergies and infections to 1.7 for fatigue, diabetes, and obesity (174).

Main factors that contribute to the delay in the diagnosis of PsA are lack of awareness among patients of the relationship between skin disease and joint symptoms and the absence of a specific diagnostic marker. Improved PsA screening is suggested in patients with psoriasis because the validated psoriasis epidemiology screening tool identified more than 10% of registry patients who could have had undiagnosed PsA (175). Recent consensus guidelines for managing psoriasis recommend using questionnaires to screen for the presence of PsA, and Salaff et al. (176) developed a self-administered questionnaire, called Simple Psoriatic Arthritis Screening (SiPAS), which screens psoriasis patients for signs and symptoms of PsA with compared high sensibility and specificity. In a systematic review, 259 possible markers associated with the development or presence of PsA in patients with psoriasis were identified in 119 studies (177). Laboratory markers related to inflammation and bone metabolism showed a strong association (not prediction) of PsA in psoriasis, however only C-X-C motif ligand 10 reached a strong level of evidence for a positive predictive value (177), but whether these indicators are clinically useful remains to be further investigated.

Active enthesitis and synovitis could be useful to identify subclinical PsA (178). Fluorescence optical imaging may be a helpful novel tool to analyze microcirculation in psoriasis/PsA patients (179).

### Osteoporosis and Fracture

Studies have yielded inconclusive results on the association between psoriatic/PsA and bone loss (180–182), a systematic review and meta-analysis including 12 studies concluded that patients with psoriasis/PsA have an increased risk of fractures (psoriasis: OR = 1.29, 95%CI = 1.02-1.63; PsA: OR = 2.88, 95%CI = 1.51-5.48). However there is little evidence supporting the association between psoriasis and osteoporosis/osteopenia (osteoporosis, psoriasis: OR, 1.28, 95%CI 0.86-1.90; PsA: OR, 1.32, 95%CI: 0.79-2.19; osteopenia, psoriasis: OR, 1.50, 95%CI: 0.75-3.02; PsA: OR, 1.61, 95%CI: 0.67-3.85) (183). Psoriasis increased the risk of osteoporosis in patients aged ≥ 40 years in Korea (182).

## Skin

Onychomycosis was the most frequent skin comorbidity of psoriasis followed by rosacea and telangiectasia (184). Psoriatic patients with skin diseases had a worse quality of life than those without, as measured by Skindex 29, Dermatology Life Quality Index, and psoriasis disability index scores (185).

### Vitiligo

A meta-analysis including 10 case-control and cross-sectional studies found psoriasis and vitiligo are bidirectionally associated with each other: increased odds for psoriasis in vitiligo patients (summary OR: 3.43, 95% CI: 1.86-6.33) as well as elevated odds for vitiligo in psoriatic patients (summary OR: 2.29, 95% CI: 1.56-3.37) (116) (**Table 4**).

### Bullous Disease

A systematic review and meta-analysis of 12 observational studies found that the overall pooled prevalence of psoriasis was 2.4% (95% CI, 1.0-4.4) among pemphigus patients (186). Pemphigus patients face an increased risk of psoriasis (187).

Psoriasis was independently associated with an increased risk of bullous pemphigoid (188, 189), with an important risk factor of younger age, and over one-third of bullous pemphigoid cases were diagnosed in the first year after incident psoriasis (188).

### Chronic Itch

Higher itch intensity was associated with women, lower educational level, pustular psoriasis, lesions on visible or sensitive areas, palmoplantar areas, severe disease, disease duration <15 years, and no or few prior systemic treatments (190). Reszke et al. (191) firstly reported the possible associations between psoriatic pruritus and drugs administrated in various systemic conditions, including antacids, beta-blockers, angiotensin enzyme converting inhibitors, and xerosis intensity angiotensin receptor blockers.

### Palmoplantar Pustulosis

Three large population-based cohorts reported that the prevalence of psoriasis was between 14.2% and 61.3% in patients with palmoplantar pustulosis, and patients both with palmoplantar pustulosis and psoriasis had a higher PsA prevalence and antipsoriatic drug use (192).

### Allergy

A positive association was reported between psoriasis and contact allergy (193), while an inverse association was reported in psoriasis and atopic diseases (194). The polarization of the activated immune response by allergens may affect the occurrence and significance of allergies in underlying immune-mediated diseases, even beyond the skin (193).

## Reproductive System

### Male

Two comprehensive meta-analyses summarized that psoriasis was associated with an increased risk of erectile dysfunction (OR, 1.22; 95% CI 1.08-1.37; P = 0.002 (195); OR, 1.62, 95%CI: 1.37-1.91, P < 0.001 (196)), especially in men with mild psoriasis (1.13; 1.09-1.20) and severe psoriasis (1.17; 1.04-1.32) (197). A pilot study supported a correlation between hypogonadism and obesity in male psoriasis patients, while no psoriasis-specific reduction of testosterone can be assumed (198).

A comprehensive literature review and meta-analysis including 28 studies found that the prevalence of sexual dysfunction (SD) ranged from 40.0% to 55.6% in psoriasis patients (199). Physical and psychological comorbidities are risk factors for SD, and the strongest association with SD is anxiety and depression, PsA, and genital psoriasis. Biologic drugs have benefits for the improvement of SD (199).

### Female

A meta-analysis including 16 studies found that pregnant women with psoriasis had a significantly higher risk of adverse maternal outcomes [preterm birth: 1.32 (1.15, 1.52); caesarean delivery: 1.33 (1.17, 1.52); (pre)eclampsia: 1.28 (1.14, 1.43); gestational hypertension: 1.30 (1.18, 1.44); gestational diabetes: 1.19 (1.10, 1.30)], but not adverse neonatal events (200).

## Respiratory System

Psoriasis and asthma might have a bidirectional association. A meta-analysis including six studies indicated that the patients with psoriasis had a higher risk of asthma susceptibility (OR 1.32 [95% CI, 1.20-1.46]), especially among the older patients (≥50 years) (201). A cohort study in Korean children found asthma was associated with the elevated risk of psoriasis (adjusted HR = 1.19; 95% CI, 1.07-1.33) (202). Patients with psoriasis who do not smoke may not have an increased risk of developing chronic obstructive pulmonary disease in US adults (203), so counselling psoriasis patients on the benefits of smoking cessation remains a valuable approach for preventing chronic obstructive pulmonary disease risk.

## Oral Comorbidities

### Geographic Tongue

GT has been described as a predictor of psoriasis, however, reports are inconclusive. A systematic review and meta-analysis including 11 case-control studies found that the frequency of GT was statistically associated with psoriasis (pooled OR was 3.53, 95% CI: 2.56-4.86) (204), however prevalence rates vary from 0.51% to 11.43% and data for Europe are sparse (205). A hospital-based cross-sectional study in Saudi Arabia found a positive association of both GT and fissured tongue in adult patients with psoriasis compared with those without (206). The PASI score was statistically higher in patients affected by GT, and exhibited less improvement after treatment (204). However a prospective case-control study in an Austrian cohort found that psoriasis was associated with fissured tongue but not with GT (207), and the severity of the disease evaluated by the PASI scale did not influence mucosal involvement (208).

### Periodontitis

A meta-analysis including two cohort studies and three case-control studies found that patients with periodontitis had a significantly elevated risk of psoriasis (pooled RR is 1.55, 95% CI, 1.35-1.77) (209). Vice versa, studies confirmed a significant psoriasis-associated increased risk of periodontitis (210–212), which was highest in patients with severe psoriasis and PsA (210). Severity of psoriasis also presented a strong relationship with periodontal clinical parameters (211). A link was identify between the inverse type of psoriasis and periodontitis (212).

## Ocular Comorbidities

Studies on ocular comorbidities of psoriasis are mainly from Taiwan. A retrospective cohort study concluded that psoriasis was associated with an increased risk of keratopathy in patients without preexisting prominent corneal disease; age older than 60 years and dry eye disease were risk factors of developing keratopathy (213). The risk of retinal diseases was significantly higher in psoriasis, including retinal detachment, retinal vascular occlusion, and retinopathy (214), and an increased risk of uveitis (215). Ocular morbidity was increased with increasing duration and PASI score (216), so it is important to screen psoriasis patients to prevent sight-threatening complications.

## Rare Comorbidities

An association was also confirmed between psoriasis and hearing loss (217), Behçet’s disease (218), scabies infection (219), and pediatric infection (220).

## Conclusion

With the increase of epidemiologic and basic scientific evidence, the nature of psoriasis is recognized as a systemic inflammatory disease. The pathogenesis of systemic inflammation of psoriasis is the development basis of extracutaneous comorbid diseases, and can explain why therapies for psoriasis including traditional systematic agents and biologics benefit the extracutaneous comorbidities of psoriasis.

In addition to CAD, IBDs, and CKD, which are recognized major comorbidities of psoriasis, more systematic diseases, such as bullous disease, asthma, and periodontitis, are identified as new comorbidities of psoriasis, and it helps to complete the understanding of psoriasis and comorbid diseases. Mental health becomes a new hot area of psoriasis study, as psoriasis not only causes a negative impact on psychological health in patients, which leads to a range of psycho-emotional consequences, but also causes substantial pathological changes of the nervous system by immune dysregulation-mediated inflammation. Bidirectional associations are proved between psoriasis and extracutaneous comorbidities, such as depression, CKD, celiac disease, and vitiligo, and it provides epidemiological evidence that psoriasis treatment benefits comorbidities.

Based on the association and pathogenesis research, biochemical indicators and technical means including artificial intelligence technology that can be used as disease evaluation and prediction indicators have entered a stage of rapid development, which may facilitate the management of diseases. However, the clinical application of these assessments and predictors requires the strong support of studies with large sample sizes.

Although the association between increasing extracutaneous comorbidities and psoriasis has been identified, the correlation between certain diseases and psoriasis is still contradictory, even when using the conclusions of systematic review and meta-analyses, which needs to be confirmed by more high-quality studies included.

Malignancy risk for biologics treatment of psoriasis draws much attention, especially in the era of the COVID-19 pandemic. however the conclusions of related studies remain conflicting. Therefore, patients receiving biologics treatment should be closely monitored.

With the in-depth study of psoriasis and its comorbidities, including epidemiological and pathological basic research and genetic molecular research, the traditional system for disease patterns is likely to be broken, therefore it is critical for both clinicians and patients to recognize the potentially risk of important comorbidities associated with psoriasis, which leads to a great disease burden for people and society. Apart from the disease itself, socio-economic and cultural factors, such as sex, age, income, and education level, all possibly act as risk factors for comorbidities, so, increased awareness of psoriasis comorbidities is critical to the management of psoriasis.

## Author Contributions

JB draft the manuscript, RD made the literature searching, LZ and XC performed the selection of articles, ES managed the program and reviewed the manuscript. All of the authors approved the submission of the current manuscript.

## Conflict of Interest

The authors declare that the research was conducted in the absence of any commercial or financial relationships that could be construed as a potential conflict of interest.

## Publisher’s Note

All claims expressed in this article are solely those of the authors and do not necessarily represent those of their affiliated organizations, or those of the publisher, the editors and the reviewers. Any product that may be evaluated in this article, or claim that may be made by its manufacturer, is not guaranteed or endorsed by the publisher.
